# The impact of artificial intelligence on the reading times of radiologists for chest radiographs

**DOI:** 10.1038/s41746-023-00829-4

**Published:** 2023-04-29

**Authors:** Hyun Joo Shin, Kyunghwa Han, Leeha Ryu, Eun-Kyung Kim

**Affiliations:** 1 Department of Radiology, Research Institute of Radiological Science and Center for Clinical Imaging Data Science, Yongin Severance Hospital, Yonsei University College of Medicine, 363, Dongbaekjukjeon-daero, Giheung-gu, Yongin-si, Gyeonggi-do 16995 South Korea; 2 Center for Digital Health, Yongin Severance Hospital, Yonsei University College of Medicine, 363, Dongbaekjukjeon-daero, Giheung-gu, Yongin-si, Gyeonggi-do 16995 South Korea; 3 Department of Radiology, Severance Hospital, Research Institute of Radiological Science and Center for Clinical Imaging Data Science, Yonsei University College of Medicine, 50-1 Yonsei-Ro, Seodaemun-Gu, Seoul 03722 South Korea; 4 Department of Biostatistics and Computing, Yonsei University Graduate School, 50-1 Yonsei-Ro, Seodaemun-Gu, Seoul 03722 South Korea

**Keywords:** Outcomes research, Radiography

## Abstract

Whether the utilization of artificial intelligence (AI) during the interpretation of chest radiographs (CXRs) would affect the radiologists’ workload is of particular interest. Therefore, this prospective observational study aimed to observe how AI affected the reading times of radiologists in the daily interpretation of CXRs. Radiologists who agreed to have the reading times of their CXR interpretations collected from September to December 2021 were recruited. Reading time was defined as the duration in seconds from opening CXRs to transcribing the image by the same radiologist. As commercial AI software was integrated for all CXRs, the radiologists could refer to AI results for 2 months (AI-aided period). During the other 2 months, the radiologists were automatically blinded to the AI results (AI-unaided period). A total of 11 radiologists participated, and 18,680 CXRs were included. Total reading times were significantly shortened with AI use, compared to no use (13.3 s vs. 14.8 s, *p* < 0.001). When there was no abnormality detected by AI, reading times were shorter with AI use (mean 10.8 s vs. 13.1 s, *p* < 0.001). However, if any abnormality was detected by AI, reading times did not differ according to AI use (mean 18.6 s vs. 18.4 s, *p* = 0.452). Reading times increased as abnormality scores increased, and a more significant increase was observed with AI use (coefficient 0.09 vs. 0.06, *p* < 0.001). Therefore, the reading times of CXRs among radiologists were influenced by the availability of AI. Overall reading times shortened when radiologists referred to AI; however, abnormalities detected by AI could lengthen reading times.

## Introduction

Artificial intelligence (AI) has been widely utilized for research in radiology, and with the emergence of commercial AI software, more efforts have been made to demonstrate the efficacy of AI software in actual practice because of clinical necessity^1–3^. Research has focused on the impact of AI on patient management and the decision-making process of doctors, in addition to the achievement of reasonable diagnostic performance using AI^2^. For radiologists, questions of interest are whether AI assistance can help prioritize images for reading, reduce missing cases, or affect reading times^2,4,5^.

Recent studies have demonstrated better diagnostic performance with AI when reprioritizing brain computed tomography (CT) for the detection of hemorrhage^6,7^. Integration of AI into mammography has been found to enhance the diagnostic performance of radiologists without increasing reading time^8^. A similar tendency was observed in the detection of bone fractures using radiographs^9,10^. Several studies have also tried to demonstrate how AI affects the reading times for chest radiographs (CXRs) or CT among radiologists^11–13^. However, most of these past studies were retrospective studies performed by simulating the clinical process or only with selected cases and radiologists in a prospective manner.

CXRs are the most commonly performed imaging studies; however, timely interpretation of CXRs by radiologists, especially for those containing critical lesions, is difficult in hospitals. Most clinicians in outpatient clinics or the emergency room (ER) frequently interpret CXRs on their own before receiving official reading reports. Due to this situation, the application of AI for CXR has attracted more attention from researchers, and the development of commercially available AI software has widely been for CXRs^1,14^. For radiologists, whether the utilization of AI during the interpretation would affect their workload is of particular interest. Concerning the reading time of radiologists, there could be a concern as to whether referring to AI results would increase workload by adding working steps or reduce decision-making time as an effective computer-assisted diagnosis system^4^. To our knowledge, few studies have demonstrated how AI actually affects reading time in real clinical situations.

Therefore, this prospective observational study aims to observe how AI affects the actual reading times of radiologists in the daily interpretation of CXRs in real-world clinical practice. In this study involving 11 radiologists and 18,680 CXRs, total reading times significantly shorten with AI use, particularly when no abnormality is detected by AI. However, if any abnormality is detected by AI, reading times do not differ between AI use and no AI use. Our findings inform that the availability of AI influences the reading times of CXRs among radiologists and that AI integration can overall shorten reading times. However, it is important to note that abnormalities detected by AI may lengthen reading times.

## Results

### Subjects and CXRs

**Fig. 1:**
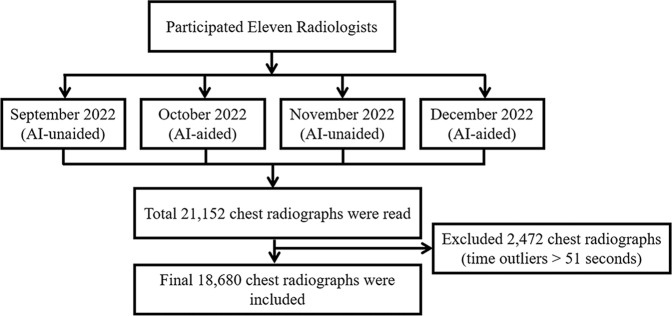
Flow diagram of the study process. Flow diagram of the study process is summarized.

During the study period, a total of 11 radiologists participated in this prospective study, and they accounted for approximately 79% of the radiologists in our institution. All radiologists who participated in the study were board-certified specialists in radiology. The participating radiologists had a minimum of 10 years and a maximum of 23 years of experience in the field of radiology. The flow diagram of the study process is summarized in Fig. 1. The data are provided in Supplementary information. The subspecialties of the participating radiologists were as follows: thoracic radiology = 1, abdominal radiology = 4, neuroradiology = 2, musculoskeletal radiology = 2, breast and thyroid radiology = 1, and health check-up = 1.

During the study period, a total of 21,152 consecutive CXRs were read by the radiologists. Among them, 2472 CXRs were excluded due to reading time outliers of 51 s according to the interquartile range (IQR) methods. Therefore, a total of 18,680 CXRs were finally analyzed. A comparison of the total number of included CXRs and the age of patients in AI-unaided and AI-aided periods is summarized in Table 1. Among the included CXRs, 9109 CXRs (49%) were read in the AI-aided period. Patient age was significantly lower in the AI-aided period (mean 57.9 years vs. 59.2 years, *p* < 0.001), and the proportion of outpatient clinic patients was higher in the AI-aided period (51.6% vs. 45.1%, *p* < 0.001). The number of CXRs containing abnormalities was significantly lower in the AI-aided period (37.4% vs. 44.5 %, *p* < 0.001).

**Table 1:** Total number of chest radiographs in AI-unaided and AI-aided conditions.

Variable	Overall (*n* = 18680)	AI-unaided (*n* = 9571)	AI-aided (*n* = 9109)	*p*-value
*Sex*				0.256
Female	9240 (49.5)	4695 (49.1)	4545 (49.9)	
Male	9440 (50.5)	4876 (50.9)	4564 (50.1)	
Age (year)*	58.52 (19.11)	59.15 (19.20)	57.85 (18.99)	<0.001
*Clinics*				<0.001
Inpatient	9658 (51.7)	5250 (54.9)	4408 (48.4)	
Outpatient	9022 (48.3)	4321 (45.1)	4701 (51.6)	
*Patient location*				<0.001
Outpatient clinic	9022 (48.3)	4321 (45.1)	4701 (51.6)	
ER	2603 (13.9)	1373 (14.3)	1230 (13.5)	
General ward	5673 (30.4)	3042 (31.8)	2631 (28.9)	
ICU	1382 (7.4)	835 (8.7)	547 (6.0)	
*Presence of previous comparable CXRs*				0.001
Absent	6940 (37.2)	3444 (36.0)	3496 (38.4)	
Present	11740 (62.8)	6127 (64.0)	5613 (61.6)	
*Atelectasis*				<0.001
Absent	16,722 (89.5)	8472 (88.5)	8250 (90.6)	
Present	1958 (10.5)	1099 (11.5)	859 (9.4)	
*Cardiomegaly*				<0.001
Absent	16,493 (88.3)	8364 (87.4)	8129 (89.2)	
Present	2187 (11.7)	1207 (12.6)	980 (10.8)	
*Consolidation*				<0.001
Absent	13,259 (71.0)	6513 (68.0)	6746 (74.1)	
Present	5421 (29.0)	3058 (32.0)	2363 (25.9)	
*Fibrosis*				<0.001
Absent	15,866 (84.9)	7979 (83.4)	7887 (86.6)	
Present	2814 (15.1)	1592 (16.6)	1222 (13.4)	
*Nodule*				<0.001
Absent	16,066 (86.0)	8088 (84.5)	7978 (87.6)	
Present	2614 (14.0)	1483 (15.5)	1131 (12.4)	
*Pleural effusion*				<0.001
Absent	15651 (83.8)	7876 (82.3)	7775 (85.4)	
Present	3029 (16.2)	1695 (17.7)	1334 (14.6)	
*Pneumoperitoneum*				0.637
Absent	18,494 (99.0)	9472 (99.0)	9022 (99.0)	
Present	186 (1.0)	99 (1.0)	87 (1.0)	
*Pneumothorax*				<0.001
Absent	18,086 (96.8)	9208 (96.2)	8878 (97.5)	
Present	594 (3.2)	363 (3.8)	231 (2.5)	
*Total abnormality scores*				<0.001
Low (<15%)	11,007 (58.9)	5308 (55.5)	5699 (62.6)	
High (≥15%)	7673 (41.1)	4263 (44.5)	3410 (37.4)	

Note. Values are presented as the total number of CXRs and a percentage in parentheses.

*Value represents a mean and standard deviation in parenthesis.

*AI* artificial intelligence, *CXR* chest radiograph, *ER* emergency room, *ICU* intensive care unit.

### Comparison of reading times according to patient characteristics

**Fig. 2:**
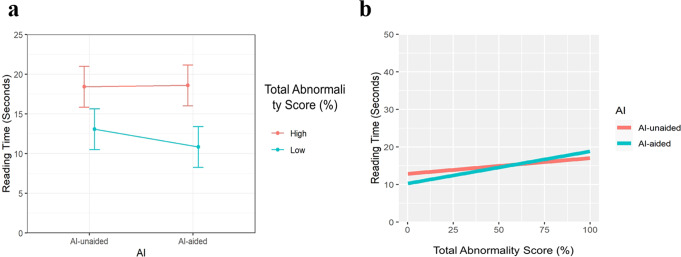
Reading time in AI-unaided and aided conditions. **a** According to the presence or absence of a lesion on chest radiographs (total abnormality scores—high: ≥15% representing the presence of any lesion, low: <15% representing the absence of any lesion by AI) and **b** according to the total abnormality scores (0–100%).

A comparison of reading times between AI-unaided and AI-aided conditions according to patient characteristics is summarized in Table 2. Total reading times were significantly shortened with the use of AI compared to no use (estimated mean 13.3 s vs. 14.8 s, *p* < 0.001) (Fig. 2a). The sex and age of patients did not affect reading times significantly (*p* = 0.108 and 0.774, respectively). Among the inpatient and outpatient clinics, reading times for outpatients significantly decreased more than those for inpatients with the use of AI (decrement −1.8 s in outpatient clinics vs. −0.5 s in inpatient locations, *p* < 0.001) (Table 2). Reading times were significantly different according to patient location (*p* < 0.001). Reading times were significantly lower with AI use when patients were in outpatient and general ward locations (*p* < 0.001 and 0.002, respectively). The presence of a previous comparable CXR did not affect reading times (*p* = 0.524) (Table 2).

**Table 2:** Comparison of reading times according to patient characteristics.

Variable	AI-unaided (s)	AI-aided (s)	Time difference in seconds (AI-aided–AI-unaided)	*p*-value	*p*-value for interaction
*Sex*					0.108
Female	14.372 (11.905, 16.84)	12.575 (10.108, 15.043)	−1.797 (−2.216, −1.378)	<0.001	
Male	15.231 (12.763, 17.699)	13.919 (11.451, 16.386)	−1.312 (−1.729, −0.895)	<0.001	
Age	0.125 (0.112, 0.138)	0.128 (0.114, 0.141)	0.002 (−0.013, 0.018)	0.774	0.774
*Clinics*					<0.001
Inpatient	15.569 (13.02, 18.118)	15.027 (12.477, 17.578)	−0.542 (−0.96, −0.124)	0.011	
Outpatient	14.246 (11.697, 16.794)	11.906 (9.358, 14.453)	−1.799 (−2.389, −1.208)	<0.001	
*Patient location*					<0.001
Outpatient clinic	14.27 (11.743, 16.798)	11.915 (9.389, 14.441)	−2.355 (−2.771, −1.94)	<0.001	
ER	14.325 (11.773, 16.877)	13.753 (11.196, 16.31)	−0.572 (−1.347, 0.203)	0.148	
General ward	16.432 (13.896, 18.967)	15.553 (13.015, 18.091)	−0.879 (−1.422, −0.336)	0.002	
ICU	15.375 (12.782, 17.968)	16.346 (13.717, 18.975)	0.971 (−0.165, 2.107)	0.094	
*Presence of previous comparable CXR*					0.524
Absent	13.622 (11.088, 16.155)	12.187 (9.653, 14.72)	1.435 (0.96, 1.911)	<0.001	
Present	15.77 (13.24, 18.299)	14.138 (11.608, 16.667)	1.632 (1.257, 2.007)	<0.001	

Note. Values are presented in time (seconds) as estimated means with 95% confidence intervals.

*AI* artificial intelligence, *CXR* chest radiograph, *ER* emergency room, *ICU* intensive care unit.

### Comparison of reading times according to the presence of lesions

Using an operating point of 15% as a cutoff value, the presence of a lesion could be determined by AI. Reading times according to the presence of lesions are summarized in Table 3. When there was no abnormality detected by AI on CXR, reading times were significantly shorter in the AI-aided period (estimated mean 10.8 s vs. 13.1 s, *p* < 0.001). However, when there was any abnormality detected by AI, reading times were not significantly different between the AI-aided and AI-unaided periods (estimated mean 18.6 s vs. 18.4 s, *p* = 0.452). The time difference between AI-aided and AI-unaided periods was significantly different according to the presence of lesions (difference of 0.2 s in the presence of any lesion vs. −2.2 s without any abnormality, *p* < 0.001) (Table 3). These tendencies were also similar for specific lesion types, except for pneumoperitoneum and pneumothorax, in terms of time differences.

**Table 3:** Comparison of reading times according to the presence of lesions.

Lesion types*	AI-unaided (s)	AI-aided (s)	Time difference in seconds (AI-aided–AI-unaided)	*p*-value	*p*-value for interaction
*Atelectasis*					<0.001
Absent	14.585 (12.124, 17.045)	12.867 (10.406, 15.327)	−1.718 (−2.027, −1.409)	<0.001	
Present	17.322 (14.816, 19.828)	17.683 (15.164, 20.203)	0.361 (−0.556, 1.279)	0.44	
*Cardiomegaly*					0.004
Absent	14.592 (12.12, 17.063)	12.912 (10.441, 15.383)	−1.68 (−1.993, −1.367)	<0.001	
Present	16.843 (14.328, 19.358)	16.504 (13.98, 19.028)	−0.339 (−1.21, 0.532)	0.446	
*Consolidation*					<0.001
Absent	14.092 (11.595, 16.588)	12.169 (9.672, 14.665)	−1.923 (−2.266, −1.581)	<0.001	
Present	17.623 (15.112, 20.133)	17.534 (15.019, 20.049)	−0.089 (−0.647, 0.469)	0.755	
*Fibrosis*					0.014
Absent	14.383 (11.907, 16.859)	12.749 (10.273, 15.225)	−1.634 (−1.953, −1.316)	<0.001	
Present	17.652 (15.145, 20.159)	17.056 (14.539, 19.573)	−0.596 (−1.36, 0.168)	0.126	
*Nodule*					<0.001
Absent	14.56 (12.099, 17.02)	12.75 (10.29, 15.211)	−1.809 (−2.124, −1.494)	<0.001	
Present	16.99 (14.497, 19.482)	17.606 (15.102, 20.109)	0.616 (−0.176, 1.408)	0.127	
*Pleural effusion*					<0.001
Absent	14.529 (12.061, 16.996)	12.73 (10.263, 15.197)	−1.799 (−2.117, −1.48)	<0.001	
Present	17.271 (14.773, 19.769)	17.348 (14.843, 19.853)	0.077 (−0.669, 0.824)	0.839	
*Pneumoperitoneum*					0.668
Absent	14.729 (12.258, 17.199)	13.171 (10.7, 15.641)	−1.558 (−1.855, −1.261)	<0.001	
Present	21.864 (18.805, 24.923)	20.948 (17.801, 24.094)	−0.917 (−3.838, 2.004)	0.538	
*Pneumothorax*					0.071
Absent	14.752 (12.28, 17.224)	13.168 (10.696, 15.64)	−1.584 (−1.884, −1.285)	<0.001	
Present	16.93 (14.27, 19.591)	16.993 (14.254, 19.733)	0.063 (−1.702, 1.829)	0.944	
*Total abnormality scores*					<0.001
Low (<15%)	13.068 (10.501, 15.635)	10.832 (8.266, 13.399)	−2.236 (−2.603, −1.869)	<0.001	
High (≥15%)	18.421 (15.848, 20.993)	18.596 (16.021, 21.17)	0.175 (−0.281, 0.631)	0.452	

Note. Values are presented in time (seconds) as estimated means with 95% confidence intervals.

*Presence of an abnormal lesion when the abnormality score is more than 15%.

*AI* artificial intelligence.

### Comparison of reading times according to abnormality scores

When the abnormality score analyzed by AI was considered as a continuous variable, reading times significantly increased as scores increased, and a more significant increase was observed with the use of AI, compared to no use (regression coefficient 0.09 vs. 0.06 for 1 s increases, *p* < 0.001) (Table 4, Fig. 2b). These tendencies were also similar for specific lesion types, except for pneumoperitoneum and pneumothorax.

**Table 4:** Comparison of reading times according to abnormality scores.

Lesion types*	AI-unaided	AI-aided	Difference (AI-aided–AI-unaided)	*p*-value	*p*-value for interaction
Atelectasis	0.075 (0.061, 0.088)	0.112 (0.097, 0.127)	0.038 (0.018, 0.057)	<0.001	<0.001
Cardiomegaly	0.038 (0.028, 0.049)	0.061 (0.049, 0.073)	0.022 (0.008, 0.037)	0.003	0.003
Consolidation	0.044 (0.037, 0.051)	0.068 (0.061, 0.075)	0.024 (0.016, 0.033)	<0.001	<0.001
Fibrosis	0.066 (0.055, 0.076)	0.082 (0.071, 0.094)	0.017 (0.002, 0.032)	0.028	0.028
Nodule	0.095 (0.078, 0.111)	0.167 (0.149, 0.185)	0.073 (0.05, 0.095)	<0.001	<0.001
Pleural effusion	0.042 (0.033, 0.051)	0.069 (0.06, 0.078)	0.027 (0.015, 0.039)	<0.001	<0.001
Pneumoperitoneum	0.119 (0.09, 0.147)	0.122 (0.094, 0.15)	0.003 (0.036, 0.043)	0.879	0.879
Pneumothorax	0.059 (0.04, 0.078)	0.079 (0.055, 0.102)	0.02 (0.009, 0.049)	0.182	0.182
Total abnormality scores	0.064 (0.058, 0.069)	0.089 (0.083, 0.095)	0.025 (0.018, 0.033)	<0.001	<0.001

Values are presented with coefficients with 95% confidence intervals.

*Abnormality score was considered as a continuous variable.

*AI* artificial intelligence.

## Discussion

Here we report reading times in the daily CXR interpretations of 11 radiologists and include all consecutive CXRs read by radiologists during 4 months to determine whether reading times are affected by the use of AI. With increases in the work burden of radiologists, whether AI could be a potential solution for reducing fatigue and enhancing the accuracy of radiologists is an interesting topic^4^. Because CXRs are read by all radiologists in our institution under preset requirements for each month, this study design mirrored what would occur in actual practice. This is an observational study performed by simply adjusting the automatic display of AI results in the PACS by month and extracted time data using PACS log records. Radiologists could read CXRs in their daily practice with or without utilizing AI results. We found that overall reading times were affected by the use of AI and, interestingly, shortened for normal CXRs. However, reading times did not significantly differ according to AI use for CXR with abnormalities. When the abnormality score on CXR increased, reading times also increased. This could be due to radiologists reporting normal CXRs with more confidence after referring to AI results, allowing them to make faster decisions. Conversely, when there was any lesion depicted by AI, radiologists might take more time to judge the validity of the AI assessment and to report more details about the findings seen on images regardless of the accuracy of displayed AI results.

Several studies have focused on reading times according to AI use. Reading times for detecting bone fractures in radiographs tended to decrease with AI^9,15^. For mammography, studies have shown conflicting results, with reading times not being significantly affected by the use of AI^16^ or decreasing up to 22.3% when AI results are available^17^. In a study by Lee et al., reading times were affected by the experience levels of radiologists even with AI, as general radiologists showed longer reading times; breast radiologists did not show any change in reading times with AI use^8^. Interestingly, a study by Pacile et al. reported results for mammography that were similar to the findings seen in this study^18^. According to the AI score in mammography, reading times decreased with lower scores and increased with higher scores representing the probability of malignancy. Authors suggested that AI results could help radiologists save time with normal mammograms by reassuring them that they had made the right judgment call and instead enabling them to focus more on images with suspicious findings^18^.

For CXR, Sung et al. performed a retrospective study with a randomized crossover design including 228 CXRs interpreted by 6 radiologists^11^. They demonstrated that the mean reading time was reduced from 24 ± 21 s to 12 ± 8 s with AI. They suggested that the relatively lower false-positive results of commercially available AI software could reduce reading times and that this impact was bigger than the risk of increasing reading times by unnecessary false-positive findings^11^. A recent multicenter study by Kim et al. used the same software as we did and demonstrated the actual influence of AI on reading times for a health screening cohort^12^. They reviewed the readings of the radiologists for all CXRs taken during 2 months with or without integration of AI on PACS. They reported a concordance rate of 86.8% between the reports made by AI and radiologists and found the median reading time to increase from 14 to 19 s with AI^12^. In a subgroup analysis, reading times increased for normal CXRs but decreased for abnormal CXRs. This result contradicts our own, which may be due to differences in the study cohort and the proportion of normal CXRs between the health screening center and our general hospital. In addition, our study utilized the most recent version of AI software, which could detect a total of eight lesions and displayed a contour map, abbreviations, and abnormality scores for each lesion on the analyzed images^1,19,20^. The software used in the study by Lee et al. could detect three kinds of lesions, including nodules, consolidation, and pneumothorax, without displaying separate abbreviations or scores for the detected lesions. This could have resulted in the different tendencies for reading times as our study additionally analyzed the influence of each lesion type and abnormality scores.

There are several limitations to this study. First, this study only utilized one source of commercially available software and the generalizability of its results could be limited. However, because our hospital integrated the AI-based lesion detection software for all CXRs and the processes for referring AI results are well organized, this could be an advantage when proving the actual influence of AI on workflow efficiency. Second, the number of CXRs containing lesions was different in the AI-unaided and aided periods unexpectedly because we did not control CXR types for participants in this observational study. One possible explanation is that the participating radiologists may have been able to read a greater number of easy and normal CXRs in the AI-aided period than in the AI-unaided period using total abnormality scores visualized on the worklist. The involved radiologists might preferentially read CXRs with low AI scores during the AI-aided period. Another possibility is that the radiologists not participating in this study could read normal CXRs more and fast in the AI-unaided period than participating radiologists using the sorting function of scores on the worklist. However, it was impossible to control CXR images containing similar proportions of each lesion during the 4-month study period, and whether radiologists prefer to read normal CXRs using the AI scoring system was not assessed in this study. Third, we could not check whether the participating radiologists indeed referred to AI results in all CXRs or prioritized worklists according to the scores during the AI-aided period. To encourage participation and compliance in this prospective study over 4 months, we allowed radiologists to read images just as they normally did and did not force them to refer to AI results for all CXRs in the AI-aided period. However, in a recent study, radiologists of our hospital answered that they refer to the AI results in about 83% of CXRs that they read in a day^21^. Therefore, we could suggest that our study reflected the actual influence of AI on the daily interpretation of radiologists. In addition, as there was only one chest radiology specialist at our institution, it was not possible to compare the reading times between specialists and non-specialists in chest radiology. We believe that investigating whether there are differences in reading times based on the experience and expertise of radiologists will be an important area for future research following this study. At last, we did not evaluate whether the presence of lesions or the abnormality score was accurate according to the radiologists’ reports or CT images. We only utilized the AI results concerning lesion type and scores when evaluating the impact of AI software on reading times. Since this study focused on the impact of AI on reading time, we could not address the separate topic of the accuracy of the AI program’s image findings. This software is already known for its excellent diagnostic performance^12,19,22^. For example, the diagnostic accuracy for lung nodule detection was excellent by showing an area under the receiver operating characteristic curve greater than 0.9^23,24^. In addition, similar accuracy has been reported for pneumothorax or consolidation^19,25^. Additionally, in recent studies at our institution, we demonstrated the actual clinical utility of AI for CXRs and also the importance of early detection of lung cancer^20,21,26^. We agreed that whether AI had accurate results and also affected the diagnosis of actual radiologists is an important point, we expect to broaden our research to encompass whether AI influences the diagnostic performance, false recall rate, or prioritization of urgent findings and to further evaluate the actual accuracy of AI in subsequent studies.

In conclusion, this prospective observational study of real-world clinical practice demonstrated that the reading times of CXRs among radiologists were influenced by the availability of AI results. Overall reading times shortened when radiologists referred to AI, especially for normal CXRs; however, abnormalities detected by AI on CXR appeared to lengthen reading times. Therefore, AI may be able to improve the efficiency of radiologists by sparing time spent on normal images and allowing them to invest this time in CXRs with abnormalities.

## Methods

### Subjects

The Institutional Review Board (IRB) of Yongin Severance Hospital approved this prospective study (IRB number 9-2021-0106), and all participants provided written informed consent to take part in this study. Informed consent was given by the radiologists who autonomously agreed to participate in this study. Attending radiologists who agreed to have the reading times of their daily CXR interpretations collected from September to December 2021 were recruited prospectively on August 2021 (Fig. 1). Radiologists who wished to participate in the study were eligible for inclusion regardless of their experience in the field of radiology, as long as they were all board-certified radiologists and employed at the hospital during the study period and agreed to the terms. Two authors in this study were excluded from the participants to minimize bias. In our hospital, radiographs, including CXRs, are read by all radiologists regardless of subspecialty, with a minimum recommendation of 500 radiographs for each month. Therefore, radiologists were requested to read CXRs just as they would normally do in their routine daily practice, with a minimum requirement of 300 CXRs per month during the study period. They independently read CXRs freely, referring to electronic medical records or available previous images while being kept blind to their reading times.

### AI application to CXR

**Fig. 3:**
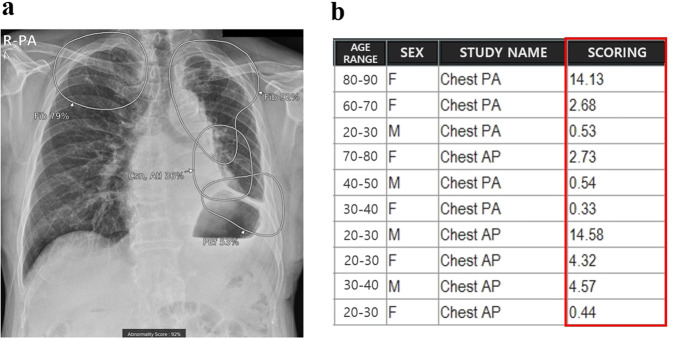
Integration of AI for CXRs on PACS. **a** The AI result attached to the second image of the original CXR contains a contour map, abbreviations, and the abnormality score of detected lesions. Doctors can simply refer to the AI results by scrolling down the original image on the PACS. **b** The highest abnormality score is used as the total abnormality score of each CXR, and this was listed as a separate column (red square) on the PACS.

In our hospital, commercially available AI-based lesion detection software (Lunit Insight CXR, version 3, Lunit, Korea) has been integrated into all CXRs since March 2020. Doctors could refer to the analyzed AI results by simply scrolling down images on the picture archiving communication system (PACS) because the analyzed results were attached to the second image of the original CXR as patients underwent examinations. The software could detect a total of eight lesions (atelectasis, cardiomegaly, consolidation, fibrosis, nodule, pleural effusion, pneumoperitoneum, and pneumothorax) and displays a contour map for lesion localization when the operating point is over 15% (Fig. 3). For detected lesions, abbreviations, and abnormality scores are displayed separately on PACS. The abnormality score represents the probability of the presence of the lesion on CXR determined by AI and ranging from 0 to 100%. Among the abnormality scores of detected lesions, the highest score was used as a total abnormality score, and this was listed as a separate column on the PACS. Therefore, doctors could refer to AI results whenever they wished, and radiologists could prioritize CXRs using the total abnormality score column on the PACS during their reading sessions if they wanted. A more detailed explanation of the integration process of AI to all CXRs was given in a recent study^20,27^. Therefore, the participating radiologists used the AI software for more than one year in the involved study period.

### Reading time measurement in AI-unaided and AI-aided periods

Reading time was defined as the duration in seconds from opening CXRs to transcribing that image by the same radiologist on the PACS. The reading time of each CXR could be extracted from the PACS log record. For the participating radiologists, we preset the PACS to not show the AI results during September and November 2021 (AI-unaided period) and to show the AI results in October and December 2021 (AI-aided period) automatically (Fig. 1). During the AI-unaided period, AI results, including secondary capture images attached to the original CXR and the abnormality score column on the worklist, were not shown on the PACS automatically, and the participating radiologists were blinded to them. However, during the AI-aided period, the results were made available and could be freely utilized by radiologists. The CXRs of patients more than 18 years old were included for analysis because the software has been approved for adult CXRs. We excluded reading time outliers with a duration of more than 51 s based on the outlier detection method. These outliers in reading time could be from various conditions, such as from delayed interpretation of corresponding CXRs after opening by unexpected interruption from other work^12^.

For the included CXRs, patient age, sex, and information on whether CXRs were taken at an inpatient or outpatient clinics were reviewed using electronic medical records. The location of patients at the time of the CXR, including the ER, general ward, and intensive care unit, was also reviewed. The presence of previous comparable CXRs was analyzed as a possible factor affecting reading times. For the AI results, the abnormality score was analyzed as both a continuous variable using the number itself and a categorical variable by applying a cutoff value of 15%. This cutoff value was chosen because our hospital has employed an operating point of 15% when determining the presence of lesions according to the vendor’s guidelines^12^. When the operating point was above 15%, the AI software marked the lesion location with a contour map, abnormality score, and abbreviation for each lesion on images^20^. Therefore, the presence of lesions, including atelectasis, cardiomegaly, consolidation, fibrosis, nodule, pleural effusion, pneumoperitoneum, and pneumothorax, were evaluated by using each abnormality score itself as a continuous variable and by applying the operating point. In addition, the highest score was used as a total abnormality score of each CXR and used to determine whether the CXRs included any abnormalities.

### Statistical analysis

For statistical analysis, the R program (4.1.3, Foundation for Statistical Computing, Vienna, Austria, package lme4, lmerTest) was used. We used the 1.5 IQR method to exclude CXRs with reading time outliers. This method is a conventional method to define outliers by using the first quartile (6 s in our study) and the third quartile (24 s). The formula to determine a cutoff value for the outlier was as follows; 24 + (24–6) × 1.5 = 51 s. The chi-square test and two-sample *t*-test were used for comparison of the total number of included CXRs and the ages of the patients in the AI-unaided and AI-aided periods. A linear mixed model was used to compare reading times considering the random effects of radiologists and patients. Reading times in seconds were compared between AI-unaided and AI-aided periods according to patient characteristics (sex, age, location, and presence of previous comparable CXR). Reading times were compared according to the presence of lesions detected by AI (any one of the following eight abnormalities: atelectasis, cardiomegaly, consolidation, fibrosis, nodule, pleural effusion, pneumoperitoneum, pneumothorax) using an operating point of 15%. When the abnormality score was considered as a continuous variable, reading times were compared between AI-unaided and AI-aided conditions. The variables, AI availability, and their interactions were considered as fixed effects for the linear mixed model. *p*-values less than 0.05 were considered statistically significant.

### Reporting summary

Further information on research design is available in the Nature Research Reporting Summary linked to this article.

## Supplementary information

Supplementary information

Reporting Summary

## Data Availability

The minimal dataset for this study is described in the Supporting Information file. The original full dataset is available upon request from the corresponding author due to its large file size.
