# Supplementary material for: The impact of artificial intelligence on the reading times of radiologists for chest radiographs
Source: NPJ Digit Med. 2023 Apr 29;6:82. doi: 10.1038/s41746-023-00829-4 (PMC10148851; doi:10.1038/s41746-023-00829-4)
Supplement: Supplementary file 1 — Supplementary information [file 41746_2023_829_MOESM1_ESM.pdf]

Supplementary Information

no	seconds	age ran	in or ou	atelecta	cardiom	consoli	fibrosis	nodule	pleural_	pneumo	pneumo	abnorm	AI	location
1	18	60-69	I	1.04	0.96	20.49	37.96	4.45	0.45	0.33	0.59	37.96	0	general
2	18	70-	I	10.2	6.93	97.51	31.09	38.36	4.01	0.49	3.42	97.51	0	general
3	6	70-	I	31.28	13.92	94.76	28.93	16.06	90.29	0.46	1.31	94.76	0	general
4	9	30-39	I	2.07	6.19	97.44	33.35	11.75	18.42	1.05	86.93	97.44	0	general
5	6	70-	I	2.94	44.41	8.8	13.39	7.13	1.49	0.4	0.31	44.41	0	general
6	6	70-	I	6.04	88.11	82.12	5.14	18.85	10.59	0.71	1.15	88.11	0	general
7	24	70-	I	27.27	28.35	96.29	28.27	10.39	95.51	0.5	1.8	96.29	0	general
8	13	60-69	I	1.32	0.24	2.19	2.58	1.71	44.8	0.43	0.47	44.8	0	general
9	8	60-69	I	0.71	0.65	2.08	0.7	1.81	0.21	0.48	0.35	2.08	0	ER
10	17	40-49	O	0.14	0.1	0.19	0.2	0.39	0.21	0.17	0.12	0.39	0	out
11	16	40-49	I	4.9	0.79	49.82	88.02	11.24	78.01	2.14	92.85	92.85	0	general
12	4	50-59	I	41.79	0.36	7.95	3.25	10.93	0.54	0.61	0.44	41.79	0	general
13	8	70-	I	2.39	0.86	12.79	5.64	4.54	1.5	1.29	2.14	12.79	0	general
14	19	70-	I	16.91	56.32	76.26	48.83	13.55	96.21	0.76	1.28	96.21	0	general
15	9	70-	I	7.22	73.17	8.56	4.43	6.9	7.76	0.45	1.61	73.17	0	general
16	40	50-59	I	75	35.19	21.87	4.23	4.18	4.3	69.08	1.51	75	0	general
17	5	70-	I	52.65	48.28	75.13	39.24	28.04	37.09	0.76	2.04	75.13	0	general
18	22	60-69	I	2.71	45.41	98.25	2.93	24.13	78.54	0.44	3.15	98.25	0	general
19	10	70-	I	18	13.69	43.03	4.87	14.76	3.35	0.4	0.7	43.03	0	ICU
20	5	50-59	I	1.77	13.36	98.51	14.24	17	9.46	0.49	0.57	98.51	0	general
21	4	70-	I	5.54	77.73	64.66	6.93	10.32	3.48	0.44	0.52	77.73	0	general
22	5	70-	I	6.94	11.66	74.5	8.5	16.73	93.09	2.35	6.49	93.09	0	general
23	7	70-	I	0.26	0.27	0.43	0.76	0.84	0.87	0.26	0.19	0.87	0	ER
24	16	70-	I	94.91	7.47	5.73	5.4	3.25	2.8	0.52	0.32	94.91	0	ER
25	30	70-	I	0.66	0.64	3.57	2.08	2.37	0.55	0.9	25.1	25.1	0	ER
26	18	70-	I	2.98	0.24	0.77	2.13	3.97	0.23	0.34	0.19	3.97	0	general
27	10	70-	I	2.22	0.33	3.67	20.56	1.94	4.82	0.49	0.37	20.56	0	general
28	13	50-59	I	0.57	0.21	1.03	1.6	40.07	0.39	0.52	0.28	40.07	0	general
29	49	50-59	I	1.11	0.32	2.47	4.98	39.03	0.36	0.48	0.32	39.03	0	general
30	4	40-49	I	1.32	0.7	49.45	70.8	47.55	1.37	0.62	0.63	70.8	0	general
31	25	70-	I	6.23	0.85	70.62	55.98	15.93	80.14	0.85	4.64	80.14	0	general
32	17	60-69	I	29.05	73.31	78.67	2.49	19.4	97.89	0.43	0.53	97.89	0	general
33	22	50-59	I	80.79	5.62	97.21	47.75	17.42	97.15	1.09	1.17	97.21	0	general
34	15	70-	I	3.09	96.71	84.45	5.25	6.89	72.32	0.55	0.55	96.71	0	general
35	6	70-	I	4.71	9.73	97.97	7.69	17.22	89.5	0.79	2.01	97.97	0	ICU
36	16	70-	I	68.96	9.34	83.93	8.91	10.09	22.16	0.8	0.66	83.93	0	ER
37	5	70-	I	11.62	37.96	66.8	4.81	6.24	25.79	0.49	1	66.8	0	ICU
38	7	70-	I	1.66	5.98	0.83	1.21	3.03	0.41	2.35	0.72	5.98	0	ER

39	9	19-29	I	0.1	0.09	0.42	0.18	0.33	0.13	0.13	0.13	0.42	0	ER
40	7	70-	I	25.49	12.78	85.23	7.59	56.43	6.12	0.68	4.03	85.23	0	general
41	5	60-69	O	0.21	1.01	0.35	7.42	0.99	0.22	0.2	0.17	7.42	0	out
42	4	50-59	O	0.23	0.14	2.66	0.53	0.87	0.33	0.27	0.23	2.66	0	out
43	6	60-69	O	0.84	0.2	1.34	1.46	1.53	7.27	0.45	0.28	7.27	0	out
44	5	60-69	O	0.21	0.28	0.74	0.8	0.88	0.77	0.24	0.19	0.88	0	out
45	6	60-69	I	40.67	1.25	76.21	16.97	9.63	5.34	5.75	9.49	76.21	0	general
46	5	60-69	O	4.04	2.66	0.42	0.31	0.7	0.25	0.25	0.1	4.04	0	out
47	5	50-59	I	1.63	3.09	98.63	3.28	58.39	4.04	0.4	0.53	98.63	0	general
48	4	60-69	O	0.27	0.2	0.29	1.41	0.78	0.25	0.21	0.15	1.41	0	out
49	4	50-59	O	0.15	0.14	0.24	1.84	0.74	0.21	0.22	0.19	1.84	0	out
50	45	70-	I	52.38	0.78	31.53	41.42	7.09	8.67	2.56	1.31	52.38	0	general
51	13	50-59	I	0.19	0.13	0.16	0.7	0.87	0.34	0.29	0.18	0.87	0	general
52	7	70-	O	0.36	1.58	0.85	0.52	1.02	0.31	0.23	0.14	1.58	0	out
53	7	70-	O	1.53	0.61	1.13	0.93	0.99	0.44	0.3	0.79	1.53	0	out
54	5	70-	O	0.92	0.22	1.07	2.51	3.35	1.38	0.41	0.22	3.35	0	out
55	20	50-59	I	0.22	0.13	0.21	1.59	0.61	0.21	0.19	0.15	1.59	0	general
56	16	70-	I	5.4	14.03	66.13	8.18	30.22	80.08	3.4	21.71	80.08	0	general
57	16	70-	O	64.8	0.55	46.99	36.92	37.17	2	0.48	0.57	64.8	0	out
58	23	70-	I	9.02	1.18	73.06	49.54	9.34	79.7	0.85	4.76	79.7	0	general
59	10	50-59	O	13.43	1.64	8.52	2.51	4.66	95.87	2.53	1.54	95.87	0	out
60	7	50-59	O	1.27	64.8	3.01	0.95	3.05	1.74	0.35	0.39	64.8	0	out
61	25	60-69	O	3.88	1.33	81.84	66.13	5.2	1.46	0.49	0.4	81.84	0	out
62	6	60-69	I	7.73	69.89	86.59	26.74	15.89	1.3	1.55	0.63	86.59	0	general
63	5	60-69	O	8.43	9.63	78.8	89.22	15.45	1.77	0.62	0.99	89.22	0	out
64	5	70-	I	13.38	59.97	94.12	4.2	15.55	89.35	0.58	1.95	94.12	0	ICU
65	19	40-49	I	14.99	1.03	23.59	90.37	8.31	59.88	1.85	54.44	90.37	0	general
66	5	70-	I	12.63	54.82	34.25	11.25	43.82	4.68	3.73	0.62	54.82	0	ICU
67	4	70-	O	1.72	22.16	5.17	2.06	3.26	0.39	0.24	0.22	22.16	0	out
68	6	50-59	O	0.39	0.44	0.56	0.62	1.42	0.38	0.24	0.22	1.42	0	out
69	19	60-69	O	93.17	0.3	9.54	23.04	3.47	2.36	1.46	0.66	93.17	0	out
70	6	70-	I	2.19	7.44	98.8	10.49	17.52	89.23	0.93	1.52	98.8	0	ICU
71	7	70-	O	1.88	3.09	97.38	60.89	10.3	38.27	0.53	6.88	97.38	0	out
72	6	70-	O	0.38	0.22	0.7	13.5	1.45	0.33	0.34	0.57	13.5	0	out
73	16	70-	I	2.1	0.57	4.31	3.06	2.76	0.75	1.81	0.47	4.31	0	ER
74	3	60-69	I	2.12	0.4	2.43	1.18	3.02	0.62	2.8	0.43	3.02	0	general
75	5	30-39	I	0.55	0.19	0.91	0.41	0.55	0.96	0.68	0.22	0.96	0	general
76	28	60-69	I	1.8	0.39	31.22	33.33	20.06	0.71	1.28	0.57	33.33	0	general
77	31	60-69	I	3.22	12.58	13.61	62.74	8.12	1.36	34.72	1.06	62.74	0	general

78	4	70-	I	0.83	28.16	3.42	1.26	6.09	0.88	0.24	0.2	28.16	0	general
79	7	50-59	I	0.27	0.14	0.33	0.45	0.55	4.65	0.27	0.18	4.65	0	general
80	6	50-59	I	0.35	0.14	0.37	3.38	0.76	0.29	0.2	0.21	3.38	0	general
81	44	30-39	I	3.59	29.98	97.02	34.74	18.31	34.22	1.25	95.1	97.02	0	general
82	9	70-	O	0.47	0.13	0.43	0.6	0.79	0.22	0.33	0.18	0.79	0	out
83	43	70-	I	12.44	7.03	79.01	20.35	15.87	72.09	1.19	1.04	79.01	0	general
84	5	70-	O	0.89	25.28	2.09	1.21	1.28	0.36	0.2	0.16	25.28	0	out
85	16	70-	I	11.96	6.34	35.05	15.6	11.69	0.84	1.77	0.59	35.05	0	general
86	51	50-59	O	50.19	0.65	16.24	13	46.19	7.66	0.46	0.35	50.19	0	out
87	5	40-49	O	60.57	0.75	31.28	18.19	4.01	3.11	1.46	0.31	60.57	0	out
88	9	60-69	I	0.43	12.4	0.85	0.38	0.56	0.3	0.27	0.15	12.4	0	general
89	5	70-	O	1.21	12.7	1.56	1.03	1.4	0.45	1.14	0.18	12.7	0	out
90	27	70-	I	2.49	8.94	89.24	4.54	17.79	1.81	0.63	0.44	89.24	0	general
91	14	70-	O	0.7	0.83	1.64	0.82	1.17	13.29	0.4	0.28	13.29	0	out
92	38	70-	O	5.86	93.72	12.01	5.48	5.94	2.38	0.21	0.21	93.72	0	out
93	8	70-	I	9.03	13.6	70.02	81.95	22.94	36.8	1.03	1.22	81.95	0	general
94	46	40-49	O	2.15	0.14	1.3	0.86	0.76	0.31	0.37	0.28	2.15	0	out
95	15	50-59	I	0.41	0.3	0.82	0.41	0.63	0.27	0.21	0.13	0.82	0	general
96	10	60-69	O	0.16	0.13	0.2	0.49	0.6	0.24	0.23	0.15	0.6	0	out
97	5	70-	I	1.68	1.38	2.19	1.84	2.44	0.32	0.27	0.19	2.44	0	general
98	28	60-69	I	9.52	38	80.28	1.87	9.19	66.1	0.79	3.72	80.28	0	ICU
99	32	40-49	I	42.86	5.03	14.88	3.79	49.82	0.43	0.62	0.38	49.82	0	general
100	7	70-	I	11.91	1.45	98.78	76.21	15.65	69.66	0.92	1.01	98.78	0	ICU
101	5	40-49	I	3.67	2.4	11.1	1.17	3.73	0.84	1.22	0.39	11.1	0	ICU
102	13	60-69	I	0.88	1.47	12.03	44.48	6.77	0.54	0.56	1.6	44.48	0	ICU
103	8	70-	I	13.62	14.99	65.79	7.03	29.98	13.97	1.37	12.39	65.79	0	ICU
104	6	40-49	O	0.21	0.13	0.27	0.58	0.93	0.19	0.19	0.13	0.93	0	out
105	8	70-	I	2.14	1.18	97.05	61.69	17.53	85.61	1.37	4.28	97.05	0	ICU
106	11	60-69	O	0.32	0.32	0.52	0.49	1.16	0.35	0.27	0.22	1.16	0	out
107	9	60-69	I	27.04	70.03	84.91	8.8	15.13	96.07	1.04	96.48	96.48	0	ICU
108	4	70-	O	0.5	2.96	0.53	2.34	1.47	0.29	0.19	0.17	2.96	0	out
109	15	50-59	I	1.29	7.29	22.31	1.28	14.41	0.65	0.45	0.65	22.31	0	ICU
110	7	70-	I	13.94	1.3	78.13	12.39	6.81	59.16	2.34	1.64	78.13	0	ICU
111	26	70-	O	3.05	85.51	2.98	2.47	3.99	7.83	0.27	0.21	85.51	0	out
112	16	60-69	I	20	14.98	10.49	23.73	10.79	12.68	1.1	3.4	23.73	0	ICU
113	14	60-69	O	20.16	0.77	28.58	68.92	19.37	90.35	1.35	56.55	90.35	0	out
114	4	70-	I	4.65	3.88	6.13	3.31	6.88	0.87	22.6	8.73	22.6	0	ICU
115	6	60-69	I	54.1	34.43	60.19	2.97	5.41	2.25	2.29	0.32	60.19	0	general
116	6	70-	O	0.27	0.12	0.33	1.76	2.59	0.33	0.56	0.21	2.59	0	out

Supplementary Figure 1. The minimal dataset data generated or analyzed during this study. The radiologists' reading times (in seconds) for each chest radiographs were recorded based on whether artificial intelligence (AI) results were available or not, along with patient age (presented in range), inpatient or outpatient status, ward type, presence of concomitant thoracic lesions as determined by AI, and AI-determined abnormality scores.
