# Supplementary material for: The impact of artificial intelligence on the reading times of radiologists for chest radiographs
Source: NPJ Digit Med. 2023 Apr 29;6:82. doi: 10.1038/s41746-023-00829-4 (PMC10148851; doi:10.1038/s41746-023-00829-4)
Supplement: Supplementary file 2 — Reporting Summary [file 41746_2023_829_MOESM2_ESM.pdf]

nature portfolio

Corresponding author(s): NPJDIGITALMED-05906R

Last updated by author(s): Mar 13, 2023

Reporting Summary

Nature Portfolio wishes to improve the reproducibility of the work that we publish. This form provides structure for consistency and transparency in reporting. For further information on Nature Portfolio policies, see our Editorial Policies and the Editorial Policy Checklist.

Statistics

For all statistical analyses, confirm that the following items are present in the figure legend, table legend, main text, or Methods section.

n/a	Confirmed	
☐	☒	The exact sample size (*n*) for each experimental group/condition, given as a discrete number and unit of measurement
☐	☒	A statement on whether measurements were taken from distinct samples or whether the same sample was measured repeatedly
☐	☒	The statistical test(s) used AND whether they are one- or two-sided<br>*Only common tests should be described solely by name; describe more complex techniques in the Methods section.*
☐	☒	A description of all covariates tested
☐	☒	A description of any assumptions or corrections, such as tests of normality and adjustment for multiple comparisons
☐	☒	A full description of the statistical parameters including central tendency (e.g. means) or other basic estimates (e.g. regression coefficient) AND variation (e.g. standard deviation) or associated estimates of uncertainty (e.g. confidence intervals)
☐	☒	For null hypothesis testing, the test statistic (e.g. *F*, *t*, *r*) with confidence intervals, effect sizes, degrees of freedom and *P* value noted<br>*Give P values as exact values whenever suitable.*
☐	☒	For Bayesian analysis, information on the choice of priors and Markov chain Monte Carlo settings
☐	☒	For hierarchical and complex designs, identification of the appropriate level for tests and full reporting of outcomes
☐	☒	Estimates of effect sizes (e.g. Cohen's *d*, Pearson's *r*), indicating how they were calculated

Our web collection on statistics for biologists contains articles on many of the points above.

Software and code

Policy information about availability of computer code

Data collection	Lunit Insight CXR, version 3, Lunit, Korea
Data analysis	R program (4.1.3, Foundation for Statistical Computing, Vienna, Austria, package lme4, lmerTest)

For manuscripts utilizing custom algorithms or software that are central to the research but not yet described in published literature, software must be made available to editors and reviewers. We strongly encourage code deposition in a community repository (e.g. GitHub). See the Nature Portfolio guidelines for submitting code & software for further information.

Data

Policy information about availability of data

All manuscripts must include a data availability statement. This statement should provide the following information, where applicable:

- Accession codes, unique identifiers, or web links for publicly available datasets
- A description of any restrictions on data availability
- For clinical datasets or third party data, please ensure that the statement adheres to our policy

All data generated or analyzed during this study are included in this published article and its Supplementary Information.

Human research participants

Policy information about studies involving human research participants and Sex and Gender in Research.

Reporting on sex and gender	Eleven radiologists read chest radiographs of 18,680 patients (M:F=9440:9240, mean 58.5 years old)
Population characteristics	During the study period, a total of 11 radiologists participated in this prospective study, and they accounted for approximately 79% of the radiologists in our institution. All radiologists who participated in the study were board-certified specialists in radiology. The participating radiologists had a minimum of 10 years and a maximum of 23 years of experience in the field of radiology. The subspecialties of the participating radiologists were as follows: thoracic radiology = 1, abdominal radiology = 4, neuroradiology = 2, musculoskeletal radiology = 2, breast and thyroid radiology = 1, and health check-up = 1.
Recruitment	Attending radiologists who agreed to have their reading times of their daily CXR interpretations collected from September to December 2021 were recruited prospectively on August 2021
Ethics oversight	The Institutional Review Board (IRB) of Yongin Severance Hospital approved this prospective study (IRB number 9-2021-0106) and all participants provided written informed consent to take part in this study.

Note that full information on the approval of the study protocol must also be provided in the manuscript.

Field-specific reporting

Please select the one below that is the best fit for your research. If you are not sure, read the appropriate sections before making your selection.

☒ Life sciences ☐ Behavioural & social sciences ☐ Ecological, evolutionary & environmental sciences

For a reference copy of the document with all sections, see nature.com/documents/nr-reporting-summary-flat.pdf

Life sciences study design

All studies must disclose on these points even when the disclosure is negative.

Sample size	Attending radiologists who agreed to have their reading times of their daily CXR interpretations collected from September to December 2021 were recruited prospectively on August 2021
Data exclusions	Radiologists who did not agree to participate in this prospective study.<br>Two authors in this study were excluded from the participants to minimize bias.
Replication	All attempts at replication were successful because the reading time of each CXR could be automatically extracted from the PACS log record.
Randomization	The participants decided to participate voluntarily in this prospective study.
Blinding	Participants independently read images freely referring to electronic medical records or available previous images while being kept blind to their reading times.

Reporting for specific materials, systems and methods

We require information from authors about some types of materials, experimental systems and methods used in many studies. Here, indicate whether each material, system or method listed is relevant to your study. If you are not sure if a list item applies to your research, read the appropriate section before selecting a response.

Materials & experimental systems

n/a	Involved in the study	
☒	☐	Antibodies
☒	☐	Eukaryotic cell lines
☒	☐	Palaeontology and archaeology
☒	☐	Animals and other organisms
☒	☐	Clinical data
☒	☐	Dual use research of concern

Methods

n/a	Involved in the study	
☒	☐	ChIP-seq
☒	☐	Flow cytometry
☒	☐	MRI-based neuroimaging
